# PHACE syndrome with severe aortic arch tortuosity: a case report

**DOI:** 10.1093/ehjcr/ytag486

**Published:** 2026-07-09

**Authors:** Yasuyuki Masuda, Kayo Ogino, Tomohiro Hayashi, Kenji Waki

**Affiliations:** Department of Pediatrics, Kurashiki Central Hospital, 1-1-1, Miwa, Kurashiki, Okayama 710-8602, Japan; Department of Pediatrics, Kurashiki Central Hospital, 1-1-1, Miwa, Kurashiki, Okayama 710-8602, Japan; Department of Pediatrics, Kurashiki Central Hospital, 1-1-1, Miwa, Kurashiki, Okayama 710-8602, Japan; Department of Pediatrics, Kurashiki Central Hospital, 1-1-1, Miwa, Kurashiki, Okayama 710-8602, Japan

**Keywords:** Neonate, Cardiovascular abnormalities, Extra-anatomical bypass, Case report

## Abstract

**Background:**

PHACE syndrome is a neurocutaneous syndrome characterized by posterior fossa malformations, haemangiomas (primarily on the face, scalp, and neck), arterial anomalies, cardiac abnormalities/coarctation of the aorta, and eye abnormalities. Cardiac abnormalities associated with PHACE syndrome typically present with coarctation of the aorta. Cases presenting with severe aortic arch tortuosity are rare.

**Case summary:**

The patient was delivered by caesarean section at 37 weeks and 0 days of gestation with a weight of 2546 g. She was diagnosed with a right aortic arch, aberrant left subclavian artery and left ductus arteriosus during the foetal period and was admitted to the neonatal intensive care unit for further evaluation. Echocardiography raised suspicion of aortic arch hypoplasia. A contrast-enhanced computed tomography showed severe aortic arch tortuosity. Cardiac catheterization performed at 3 months of age showed extremely elevated left ventricular pressure. An extra-anatomical bypass was performed at 4 months of age. A diagnosis of PHACE syndrome was made based on the combination of specific aortic arch anomalies, cerebral carotid artery anomalies, and haemangioma in the deep neck space.

**Discussion:**

In this case, severe aortic arch tortuosity detected immediately after birth resulted in stenosis. If this distinctive aortic morphology is present, PHACE syndrome should be considered in the differential diagnosis.

Learning points

- If aortic arch tortuosity is present, PHACE syndrome should be considered in the differential diagnosis.
- Multimodality imaging and invasive diagnostics evaluation are essential in the management of PHACE syndrome.

## Introduction

Frieden *et al*.^1^ first proposed the term ‘PHACE’, an acronym for posterior fossa malformations, haemangiomas, arterial anomalies, coarctation of the aorta and cardiac defects, and eye abnormalities. The exact incidence of PHACE syndrome is unknown; however more than 300 cases have been reported in the literature.^2^ Aortic arch tortuosity in PHACE syndrome is rare, with only two cases reported to date.^3^

## Summary figure

**Figure:**
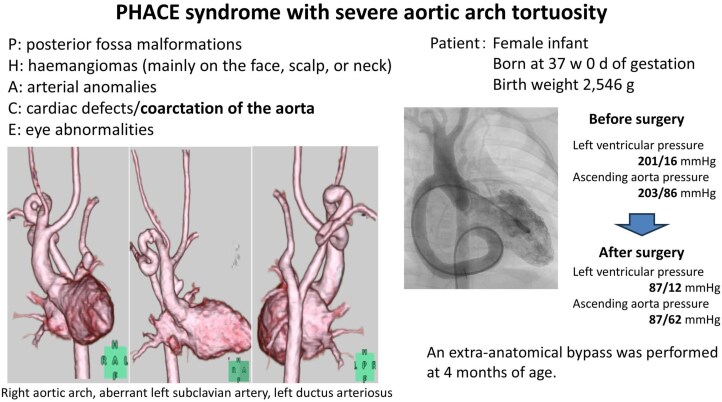


## Case presentation

Foetal echocardiography at 22 weeks of gestation revealed a right aortic arch, aberrant left subclavian artery, and a left ductus arteriosus. The patient, a female infant, was delivered by caesarean section at 37 weeks and 0 days of gestation, with a birth weight of 2546 g. Apgar scores were 8 and 9 at 1 and 5 min, respectively.

Because she had an aortic anomaly causing a vascular ring, she was admitted to the neonatal intensive care unit for imaging studies to assess tracheal stenosis and other associated conditions. Vital signs were as follows: Blood pressure was 58/34 mmHg (upper limb), and 54/44 mmHg (lower limb), heart rate was 114 beats per minute, respiratory rate was 54 breaths per minute, and oxygen saturation was 100% (room air).

A physical examination revealed clear breath sounds, regular heart sounds, no heart murmurs, and weak femoral pulses. No haemangiomas were observed. She was the first child, and there was no relevant family history. Echocardiographic findings were consistent with the foetal diagnosis. In addition, aortic arch hypoplasia was suspected, and the blood flow pattern in the abdominal aorta indicated stenosis (*Figure 1A*).

**Figure 1:**
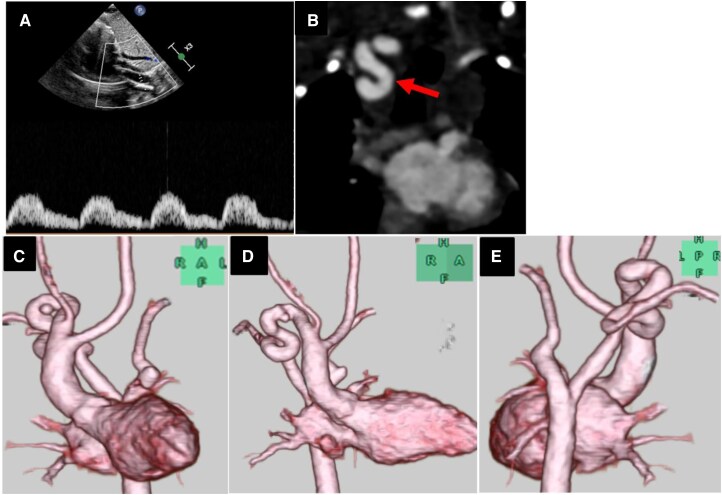
Imaging studies at Day 1. (*A*) Transthoracic echocardiography shows an abnormal blood flow pattern in the abdominal aorta, indicating stenosis. (*B*) Contrast-enhanced computed tomography shows severe tortuosity of the aortic arch (arrow). (*C–E*) Three-dimensional computed tomography shows severe tortuosity of the aortic arch.

A contrast-enhanced computed tomography performed at Day 1 revealed severe aortic arch tortuosity (*Figure 1B–E*). Because the aortic arch had a long stenotic segment, repair with a prosthesis was considered necessary. However, as there was no left ventricular hypertrophy or impaired cardiac contractility, surgical intervention was postponed until the patient had grown. Her general condition was good, and she was discharged from hospital on Day 6.

Follow-up echocardiography after discharge revealed progressive left ventricular hypertrophy, suggesting worsening aortic arch stenosis. However, the electrocardiogram did not demonstrate left ventricular hypertrophy (*Figure 2A*). As both the left and right subclavian arteries were located distal to the stenosis, it was difficult to estimate the pressure difference across the stenosis. Morphological evaluation of the stenosis using echocardiography was also challenging. Cardiac catheterization was the only method available to measure the pressure gradient.

**Figure 2:**
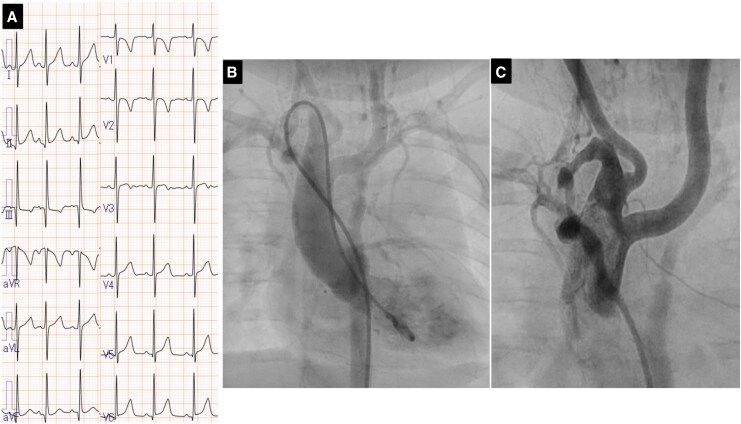
Imaging studies at 3 months of age. (*A*) The electrocardiogram does not show left ventricular hypertrophy. (*B*) Left ventricular angiography. The left ventricular pressure was 201/16 mmHg. (*C*) Retrograde angiography shows stagnation of contrast within the stenotic aortic arch, with no evidence of washout. Retrograde flow is evident in both vertebral arteries. The ascending aortic pressure was 203/86 mmHg.

Cardiac catheterization performed at 3 months of age showed markedly elevated pressures in the left ventricle (201/16 mmHg) and the ascending aorta (203/86 mmHg), with normal pressure in the descending aorta (75/69 mmHg). Retrograde angiography of the proximal aortic arch showed stagnation of contrast within the stenotic segment, with no evidence of washout. Retrograde flow in both vertebral arteries was also observed (*Figure 2B, C*; Supplementary Material online, *Videos S1* and *S2*). A contrast-enhanced computed tomography revealed progression of the stenosis compared with that on Day 1. Head magnetic resonance imaging and whole-body contrast-enhanced computed tomography were performed to assess for other vascular lesions.

These studies revealed tortuosity and meandering of the left internal carotid artery, poor visualization of the right internal carotid artery, and a haemangioma in the deep neck space (*Figure 3*), thereby meeting the diagnostic criteria for PHACE syndrome. The patient was diagnosed with PHACE syndrome.

**Figure 3:**
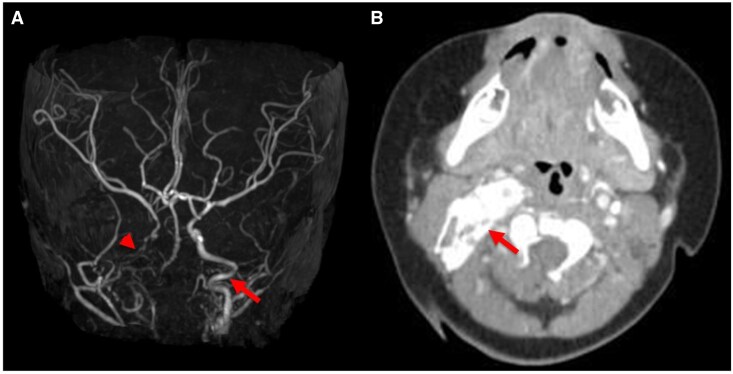
Imaging studies at 3 months of age. (*A*) Magnetic resonance imaging shows tortuosity and meandering of the left internal carotid artery (arrow) and poor visualization of the right internal carotid artery (arrowhead). (*B*) Contrast-enhanced computed tomography shows a haemangioma in the deep neck space (arrow).

At 4 months of age, an extra-anatomical bypass consisting of an ascending-to-descending aortic bypass using an 8-mm ringed ePTFE vascular graft was performed. First, a median sternotomy was carried out. Cardiopulmonary bypass was initiated with ascending aortic cannulation and right atrial drainage, and the core body temperature was reduced to 28°C. When establishing cardiopulmonary bypass, efforts were made to minimize cerebral ischaemia. Although the cooling of the lower extremities was insufficient, we had already planned to anastomose the vascular graft; therefore, it was anastomosed to the descending aorta to provide distal perfusion. Finally, while the heart was still beating, the ascending aorta was side-clamped, and the vascular graft was anastomosed to the ascending aorta.

After surgery, propranolol was initiated to treat left ventricular hypertrophy. Cardiac catheterization was performed 3 months after surgery. The left ventricular and ascending aortic pressures improved to 87/12 mmHg and 87/62 mmHg, respectively, and no stenosis was observed in the bypass graft (*Figure 4A*; Supplementary Material online, *Video S3*). An electrocardiogram did not show left ventricular hypertrophy (*Figure 4B*).

**Figure 4:**
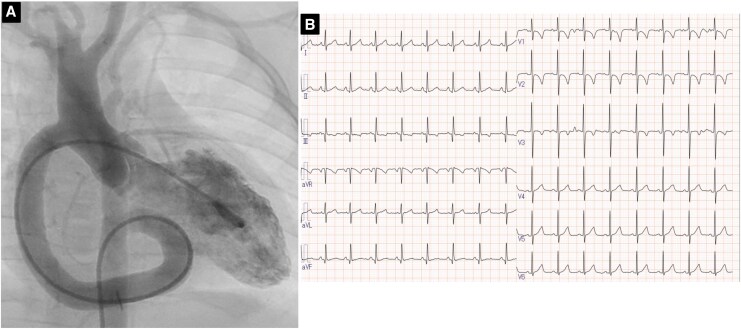
Angiograms and electrocardiogram at 3 months after cardiac surgery. (*A*) Left ventricular angiography shows no stenosis in the extra-anatomical bypass. The pressures were 87/12 mmHg in the left ventricle and 87/62 mmHg in the ascending aorta. (*B*) The electrocardiogram does not show left ventricular hypertrophy.

Propranolol treatment was discontinued at 1 year of age due to improvement in left ventricular hypertrophy. A contrast-enhanced computed tomography performed at 1 year and 6 months of age showed residual blood flow in the right aortic arch (*Figure 5*), with no new stenosis or occlusion in other vessels. The haemangioma in the deep neck space was still present but had decreased in size.

**Figure 5:**
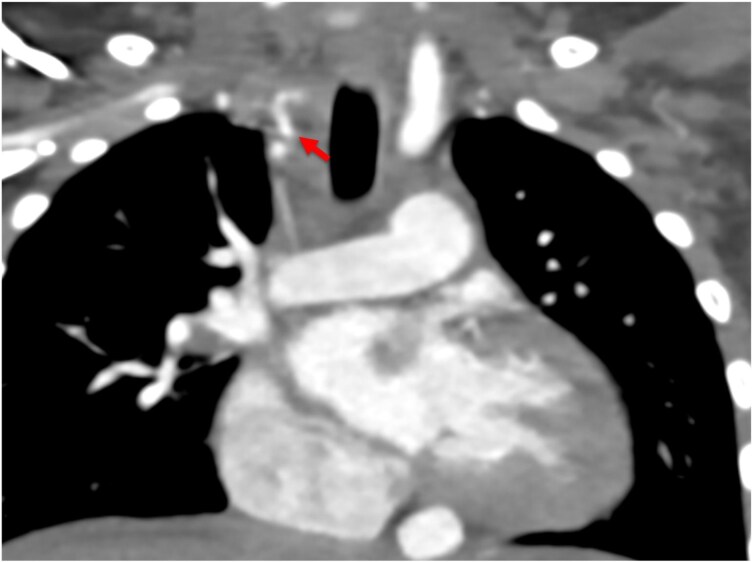
Contrast-enhanced computed tomography at 1 year and 6 months of age. Residual blood flow in the right aortic arch is present, with no new stenosis or occlusion in other vessels(arrow).

## Discussion

A definitive diagnosis of PHACE syndrome is established when an infantile haemangioma greater than 5 cm in diameter is present on the face or scalp, together with one major criterion or two minor criteria; alternatively, when an infantile haemangioma is present on the neck, upper trunk, or on the lower trunk and proximal upper extremity, together with two major criteria.^4^

Haemangiomas are a key feature of PHACE syndrome. In this case, however, the diagnosis was difficult due to the presence of a haemangioma in the deep neck.

Because few cases of PHACE syndrome with severe aortic arch tortuosity have been reported, the natural history remains largely unknown. Furthermore, to our knowledge, no reports to date have described the disease course from immediately after birth.

In this case, aortic arch tortuosity was already present at birth. As left ventricular hypertrophy was subsequently detected by echocardiography, the aortic arch tortuosity was considered to represent stenosis. Progressive stenosis is life-threatening and requires prompt medical intervention. As echocardiography revealed left ventricular that gradually worsened, cardiac catheterization was required to measure the pressure gradient and evaluate the morphology of the aortic arch. If left ventricular hypertrophy is detected, prompt evaluation by cardiac catheterization is necessary.

In this case, significant left ventricular hypertension was observed; therefore, an extra-anatomical bypass between the ascending and descending aortas was performed. The reasons for adopting this surgical technique were concerns regarding the risk of cerebral ischaemia associated with procedures involving the carotid arteries, the desire to preserve the region around the aortic arch without dissection to allow for future anatomical repair during growth, and consideration of the potential for retrograde aortic development.

The cause of PHACE syndrome with aortic arch tortuosity is unknown: however histopathological examination of affected aortic tissue from patients with PHACE syndrome has revealed loss of smooth muscle and elastin.^5^ Furthermore, several studies have demonstrated structural abnormalities in the intima and media of the arterial wall.^6–9^ In addition to structural changes in the aortic wall, haemodynamic factors such as blood flow present during the foetal stage, may also have contributed.

## Conclusion

If an infant presents with aortic arch tortuosity, PHACE syndrome should be considered in the differential diagnosis. Multimodality imaging and invasive diagnostics evaluations are essential in the management of PHACE syndrome.

## Supplementary Material

ytag486_Supplementary_Data

## Data Availability

The data underlying this article are available in the article and in its online Supplementary material.

## Lead author biography

Yasuyuki Masuda graduated from Kindai University in 2014.I have worked in Kurashiki Central Hospital since 2023.

## Notes

**Consent:** The authors confirm that written consent for the submission and publication of this case report, including images and associated text, has been obtained from the patient’s family in accordance with COPE guidelines.
