# Supplementary figures and images for: PHACE syndrome with severe aortic arch tortuosity: a case report
Source: Eur Heart J Case Rep. 2026 Jul 9;10(7):ytag486. doi: 10.1093/ehjcr/ytag486 (PMC13384066; doi:10.1093/ehjcr/ytag486)

## Slide 1
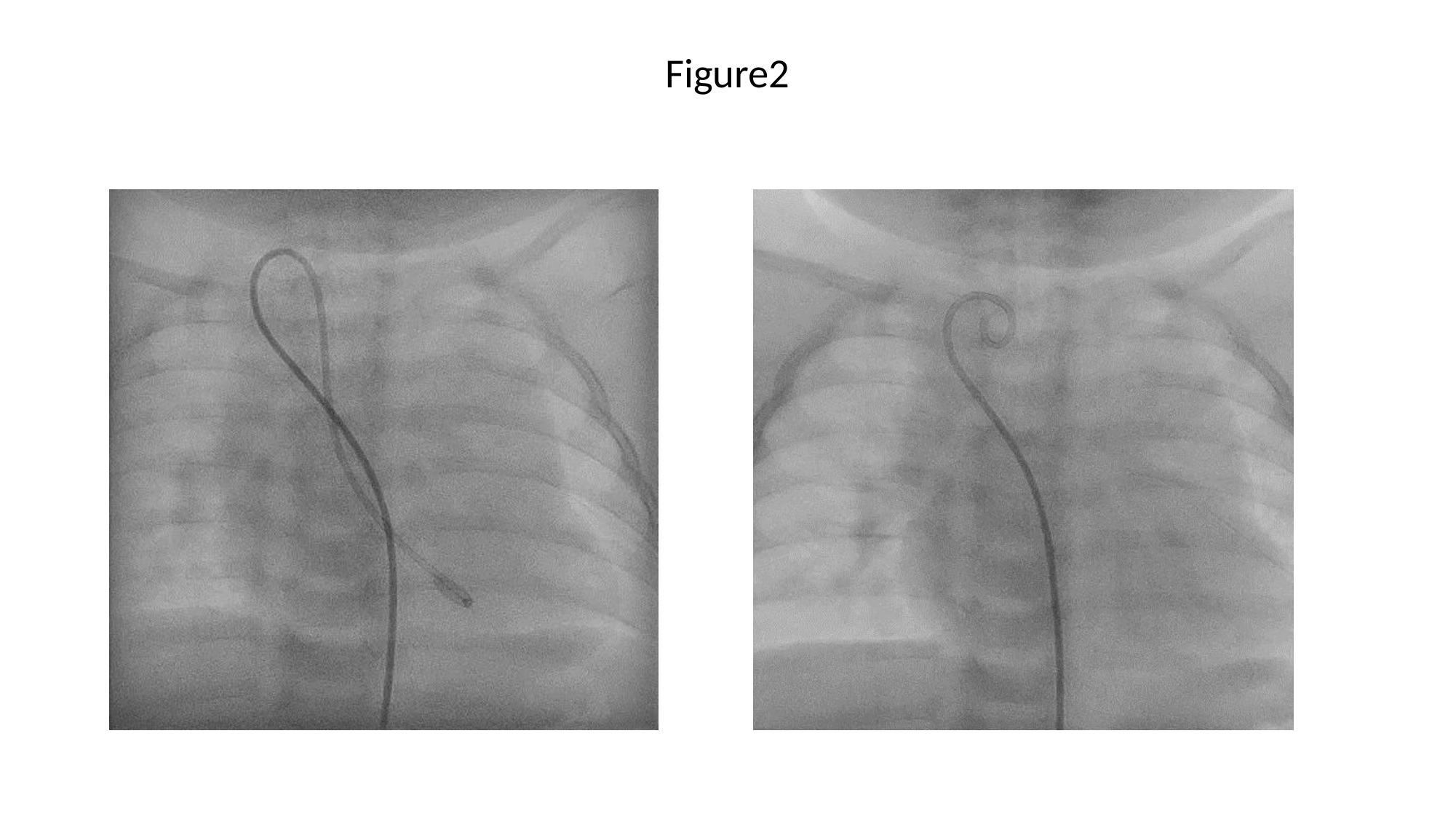

Figure2

## Slide 2
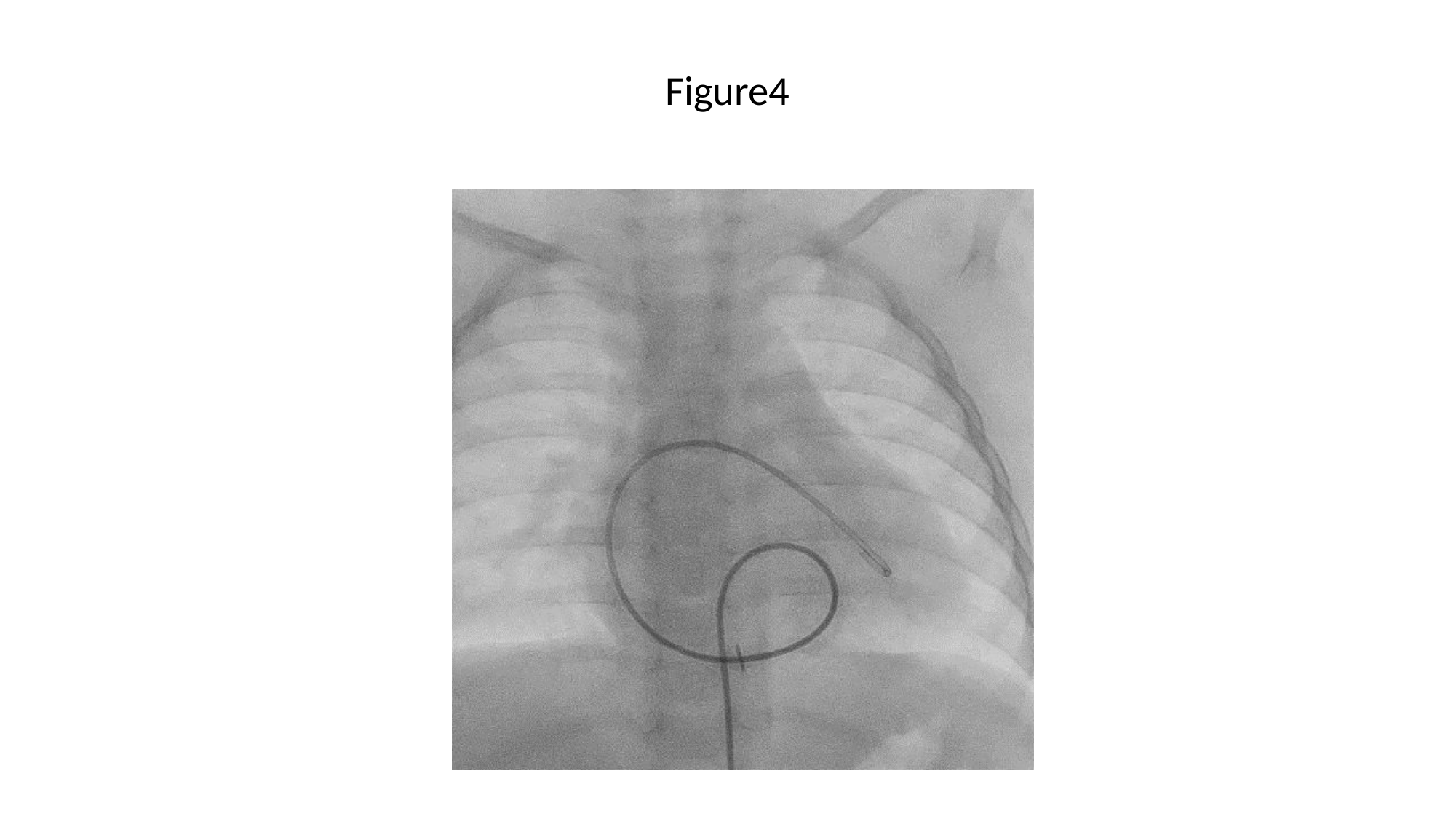

Figure4

Supplement: ytag486_Supplementary_Data [file ytag486_supplementary_data.pptx]
